# Cooled Radiofrequency Ablation Treatment of the Genicular Nerves in the Treatment of Osteoarthritic Knee Pain: 18‐ and 24‐Month Results

**DOI:** 10.1111/papr.12844

**Published:** 2019-11-14

**Authors:** Corey Hunter, Tim Davis, Eric Loudermilk, Leonardo Kapural, Michael DePalma

**Affiliations:** ^1^ Ainsworth Institute of Pain Management New York New York U.S.A.; ^2^ Orthopedic Pain Specialists Santa Monica California U.S.A.; ^3^ Piedmont Comprehensive Pain Management Group Greenville South Carolina U.S.A.; ^4^ Center for Clinical Research Winston Salem North Carolina U.S.A.; ^5^ Virginia iSpine Physicians Richmond Virginia U.S.A.

## Abstract

**Objective:**

The primary objective of this observational, prospective, multicenter study was to evaluate the long‐term outcomes, including pain, function, and perceived effect of treatment, in subjects undergoing cooled radiofrequency ablation (CRFA) who have pain due to osteoarthritis (OA) of the knee.

**Methods:**

This analysis included a subset of subjects previously enrolled in a prospective, multicenter randomized study comparing the safety and effectiveness of CRFA and intra‐articular steroid injection in patients with knee OA through 12 months who were contacted to participate in this extension study. Subjects were enrolled if they agreed to participate in up to 2 additional follow‐ups, at 18 and 24 months.

**Results:**

Eighty‐three subjects from the 5 participating sites underwent CRFA during the original study and were contacted for this extension study. Of the 33 subjects enrolled, 25 were evaluated at 18 months after CRFA treatment, and their mean numeric rating scale (NRS) score was 3.1 ± 2.7, with 12 subjects reporting ≥50% pain relief compared to baseline. At 24 months, 18 subjects reported a mean NRS score of 3.6 ± 2.8, with 11 demonstrating ≥50% pain relief. Functional improvement as measured by the Oxford Knee Score continued to be present, with an overall mean change from baseline of 26.0 ± 9.6 points at 18 months and 29.9 ± 10.4 points at 24 months.

**Conclusion:**

In this subset of subjects from a randomized controlled trial, CRFA provided sustained pain relief, improved function, and perceived positive effect through 24 months for subjects with OA knee pain with no safety concerns identified.

## Introduction

Osteoarthritis (OA) is a chronic degenerative condition that can cause substantial pain and negatively impact patient function. While total joint replacement is a well‐established treatment of last resort for late‐stage OA of the major joints, such as the hip and knee, not all patients are candidates for this procedure due to early‐stage disease, age, health, or other factors. In addition, joint arthroplasty procedures can present an increased risk for morbidity and mortality and may result in significant postoperative pain.^1^ A limited number of treatment options are available for patients who are not candidates for total joint replacement and/or for whom pharmacological therapy is either ineffective or interferes with their quality of life and general health due to serious side effects. Intra‐articular steroid (IAS) injection provides significant short‐term pain relief,^2^ but requires multiple treatments to maintain efficacy, which in turn increases the risk for serious adverse events such as septic arthritis and may exacerbate cartilage destruction.^3^, ^4^ Viscosupplementation, while showing moderate effectiveness,^3^ is not recommended in the treatment paradigm for knee OA by the American Academy of Orthopaedic Surgeons due to limited supporting data.^5^

Cooled radiofrequency ablation (CRFA) has been shown to provide at least 12 months of relief for painful conditions of the spine,^6^, ^7^, ^8^, ^9^, ^10^ and has recently emerged as a minimally invasive option for pain control in patients with OA of the knee.^11^ An initial report by Bellini suggested CRFA can provide 12 months of analgesia when used in this patient population.^12^ A prospective, multicenter randomized study involving 151 subjects with chronic knee pain (≥6 months) compared CRFA to IAS injection for pain management. Results at 6 months showed favorable outcomes, with 74% of subjects in the CRFA group having at least 50% reduction in baseline pain compared to 16% in the IAS group (*P* < 0.0001).^13^ In addition, subjects originally treated with CRFA were evaluated at 12 months postprocedure, with 65% of subjects maintaining ≥50% pain relief following a single treatment.^14^ Substantial functional improvements were also noted throughout the trial.

Given the results identified during the 12‐month analysis, it was decided to attempt to capture data describing the 18‐ and 24‐month outcomes, including pain, function, and perceived effect from subjects treated with CRFA in the original study. To our knowledge, no other study has prospectively reported the effects of RFA on OA knee pain beyond 12 months post‐treatment.

## Methods

This prospective, observational, multicenter study was conducted by extending the follow‐up period of the original prospective, randomized controlled study comparing CRFA to IAS.

In the original study, subjects were randomized 1:1 to receive either CRFA or IAS, with follow‐up visits conducted at 1, 3, 6, and 12 months.^13^ Subjects randomized to CRFA were treated using COOLIEF* cooled radiofrequency (Avanos Medical, Inc., Alpharetta, GA, U.S.A.) applied to three nerves of their OA‐affected knee (single lesion at each location). RFA generates lesions through ionic heating created by passing an electric current through a radiofrequency probe. Surrounding tissue will reach a temperature of 80°C, which causes the thermal destruction of nervous tissues.^15^ Pain is attenuated while nerve structures are healing.

Cooled radiofrequency differs from standard radiofrequency in the sense that cooled radiofrequency probes are internally cooled with water. This cooling prevents charring and insulation at the tissue–tip interface, generating both larger lesions as well as delivering significantly more energy to the surrounding tissue.^15^

Under fluoroscopic visualization, cooled radiofrequency probes were placed adjacent to the superomedial and inferomedial branches of the saphenous nerve and the superolateral branch of the femoral nerve, which were previously confirmed to be transmitting painful signals. Lesioning was performed at clinical parameters previously outlined.^13^

**Figure 1:**
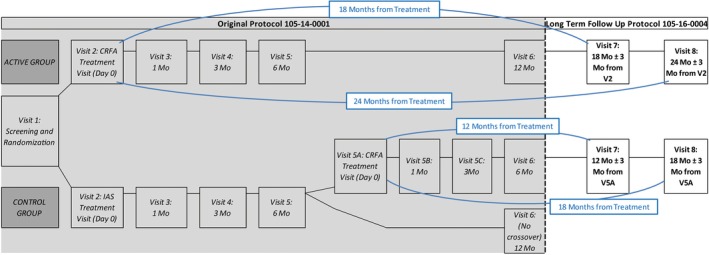
Context of 24‐month outcomes analysis within clinical trial design. CRFA, cooled radiofrequency ablation.

After the 6‐month follow‐up visit, subjects dissatisfied with the IAS treatment could cross over (XO) to CRFA. In this current analysis, patients originally randomized to CRFA were followed at 18 and 24 months after the initial treatment, while XO patients were followed up at 12 and 18 months after the CRFA treatment (Figure 1). For ease of discussion within this article, these visits will only be referred to as 18‐ and 24‐month visits, respectively. Twenty‐four‐month data presented in this report are derived from patients in this trial who were originally treated with CRFA following randomization.

The original study was conducted at 11 sites.^13^ This extension study was designed following the receipt of the 12‐month data analysis of the original study. At the time of initiation of this extension study, 2 investigators were no longer at the institutions where the original study was conducted, and those sites were unable to be considered for this exercise. Therefore, 9 of the original investigators were contacted regarding this extension analysis, and 5 elected to participate and were able to complete the required obligations for participation (contract, institutional review board [IRB], etc.). IRB approval was obtained, and subjects from these 5 sites who were previously treated with CRFA were contacted to determine their eligibility for the extension study. At least 2 attempts to contact each subject were documented.

All subjects were considered for this extension study if they were previously treated with CRFA as part of the original study, even if they did not complete the study or withdrew prematurely.^13^ Additional inclusion criteria were willingness to provide informed consent and participation in up to 2 additional follow‐ups, either via phone or in person. Subjects were excluded if they had an injury, trauma, or procedure on the index (treated) knee, since this would prevent a meaningful assessment of the effects from the CRFA procedure received as part of the initial study. However, known information related to other procedures (procedure received, date received, etc.) was maintained and is presented in Table 1. Examples of disqualifying procedures included partial or total knee replacement, IAS, arthroscopic debridement, hyaluronic acid injection, RFA, cryoablation, and platelet‐rich plasma (PRP) injection.

**Table 1:** Disqualifying Knee Procedures

Procedure Type	Number of Subjects	Days from CRFA Mean (Range)
Steroid injection	4	373 (26 to 605)
Total knee arthroplasty	4	359 (254 to 416)
Hyaluronic injection	2*	164 (160 to 168)
Repeat CRFA	2	536 (516 to 555)
Platelet‐rich plasma injection	1	315 (NA)
Arthroscopy	1	136 (NA)
Injury	1	664 (NA)
Any procedure	15	363 (26 to 664)

CRFA, cooled radiofrequency ablation; NA, not applicable.

* One subject reported a hyaluronic injection 168 days after CRFA treatment, and steroid injections 196 and 410 days after CRFA treatment. Only the earliest disqualifying procedure or injury is summarized here.

Assessments were performed for available subjects at each time point and included pain, overall function, and perceived treatment effect. Subjects were not required to attend both the 18‐ and the 24‐month visits to be included in the analysis.

### Study Outcomes

For in‐office visits (34/54 total [63%]), subjects completed questionnaires independent of study staff. For phone visits, the coordinators recorded subjects’ verbal responses. Pain was assessed utilizing an 11‐point numeric rating scale (NRS), with 0 indicating no pain and 10 indicating the worst pain ever.^16^ Patients were asked to provide their assessment of pain in the following categories: least pain, worst pain, pain right now, and usual daily pain for the past 7 days. Raw values were then utilized to calculate the mean at each time point.

The Oxford Knee Score (OKS) was used to evaluate overall knee function of subjects. The OKS is a 12‐item questionnaire about pain and function that provides a single score, ranging from 0 (most difficulties) to 48 (least difficulties).^17^

The Global Perceived Effect (GPE) scale, a quality‐of‐life outcomes instrument, was used to determine subjects’ perceptions of treatment effects from CRFA.^18^ The GPE scale used in this study included a single question: “Since your treatment, how would you rate your knee condition?” The 7‐point scale, ranging from 1 (worst ever) to 7 (best ever), was collapsed during analysis to “improved” (score of 5 to 7) and “not improved/worse” (score of 1 to 4) for ease of interpretability. All subjects were evaluated for adverse events (AEs) and serious AEs at each visit.

A Kaplan‐Meier curve was constructed to provide further insight into both the actual and censored outcomes in the study. An individual patient’s pain relief measured by the NRS falling below 50% from baseline was considered a terminal event for this analysis.

### Statistical Analysis

Data were reported using descriptive statistics, including means, standard deviations (SDs) and 95% confidence intervals (CIs) for continuous outcomes, and counts, percentages, and 95% CIs for categorical outcomes. Data from the original study^13^ were also incorporated into this analysis (ie, demographic information, treatment information, outcomes from previous visits, etc.) to allow for discussion of full patient experience post‐CRFA.

## Results

**Figure 2:**
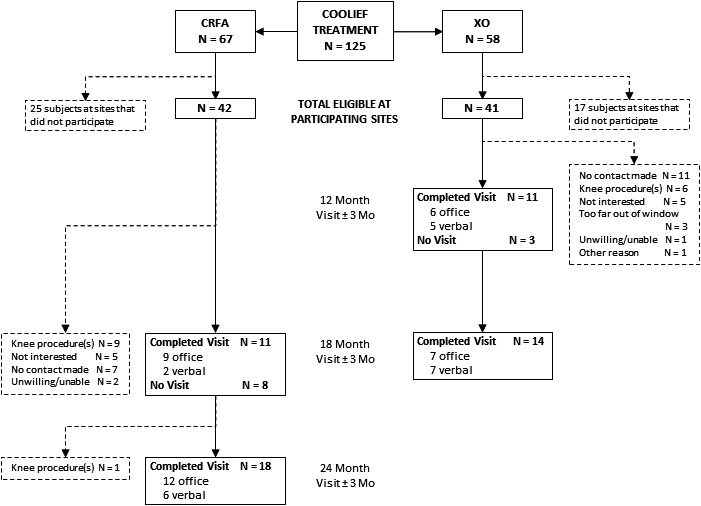
Consolidated Standards of Reporting Trials (CONSORT) diagram of study subject disposition. CRFA, cooled radiofrequency ablation.

The original study treated 125 subjects with CRFA across 11 sites.^13^ From the 5 sites participating, 83 subjects were treated in the original study, with 42 randomized to and treated with CRFA, and 41 randomized to and treated with IAS who chose to XO to CRFA after 6 months. Of these 83 available subjects, 15 (18.1%) did not qualify as a result of having another procedure (*n* = 14) or major knee trauma (*n* = 1) prior to this extension study, 35 (42.2%) could not be reached or declined participation, and the remaining 33 (40.0%) elected to participate (Figure 2).

The 33 subjects who agreed to participate in this extension study (19 original CRFA and 14 XO) provided consent, and 25 subjects were evaluated at 18 months after CRFA treatment and 18 subjects were evaluated at 24 months after CRFA treatment. One subject (3.0%) enrolled in the extension study was withdrawn after the 18‐month follow‐up evaluation due to undergoing a disqualifying knee procedure.

### Disqualifying Knee Procedures

Fifteen subjects reported receiving additional therapy since their last visit in the previous study and were therefore excluded from this extension study. As seen in Table 1, subjects reported undergoing a variety of procedures, including steroid injection (*n* = 4), total knee arthroplasty (*n* = 4), hyaluronic injection (*n* = 2), repeat CRFA (*n* = 2), PRP injection (*n* = 1), and arthroscopy (*n* = 1).

### Numeric Rating Scale

**Figure 3:**
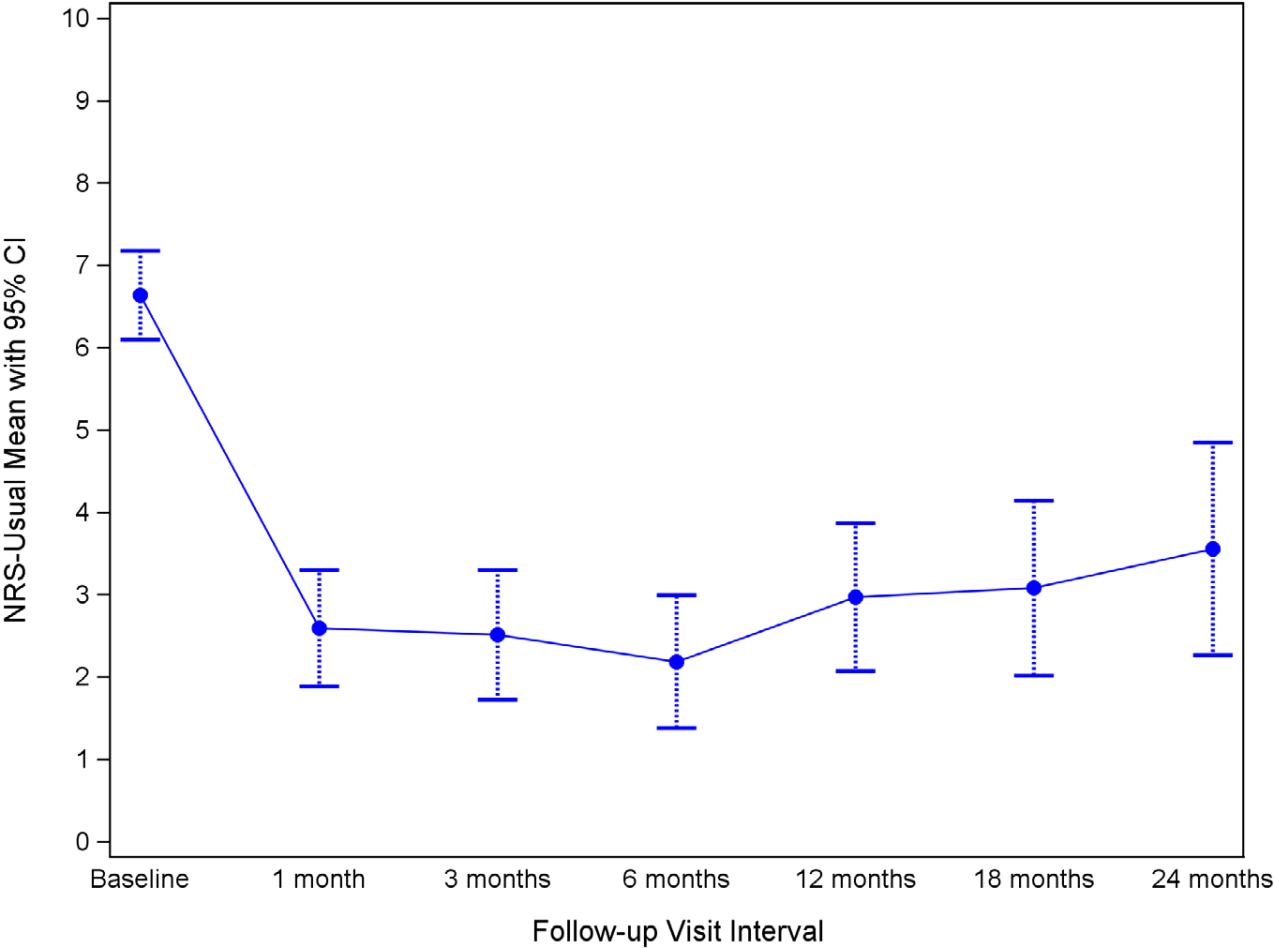
Chronology of numeric rating scale (NRS) study results. CI, confidence interval.

Pain was evaluated using the NRS based on the subject’s usual level of pain during the week prior to assessment. The mean (±SD) baseline NRS pain score for subjects treated with CRFA was significantly decreased (*P* < 0.0001), from 6.6 ± 1.6 at baseline to 3.1 ± 2.7 (*n* = 25) and 3.6 ± 2.8 (*n* = 18) at 18 and 24 months, respectively. Results in Table 2 and Figure 3 contain data specifically from the subjects included in the extension study. These results clearly demonstrate that patients can have clinically significant pain relief through 24 months following a single CRFA treatment; 12 subjects at 18 months and 11 subjects at 24 months continued to experience at least 50% reduction in pain from their baseline values.

**Table 2:** Numeric Rating Scale Results

	Baseline *n* = 33^b^	1 Month *n* = 32^b^	3 Months *n* = 31^b^	6 Months *n* = 32^b^	12 Months *n* = 30^b^	18 Months *n* = 25	24 Months *n* = 18
Numeric rating scale^a^							
Mean ± SD	6.6 ± 1.6	2.6 ± 2.0	2.5 ± 2.2	2.2 ± 2.3	3.0 ± 2.5	3.1 ± 2.7	3.6 ± 2.8
95% CI	6.1 to 7.2	1.9 to 3.3	1.7 to 3.3	1.3 to 3.0	2.0 to 3.9	2.0 to 4.2	2.2 to 4.9
Change from baseline (%)^a^							
Mean ± SD	—	60.8 ± 26.3	62.9 ± 32.9	65.9 ± 31.9	52.4 ± 39.0	50.6 ± 40.2	50.4 ± 41.0
95% CI	—	51.3 to 70.3	50.8 to 75.0	54.4 to 77.4	37.9 to 67.0	34.0 to 67.2	30.0 to 70.8
At least 50% improvement in pain							
*n*	—	23	22	26	16	12	11

a Data are presented as mean and standard deviation (SD) along with 95% confidence interval (CI).

b Data from the original study were included from baseline to 12 months for subjects enrolled in this extension study.

### Oxford Knee Score

**Figure 4:**
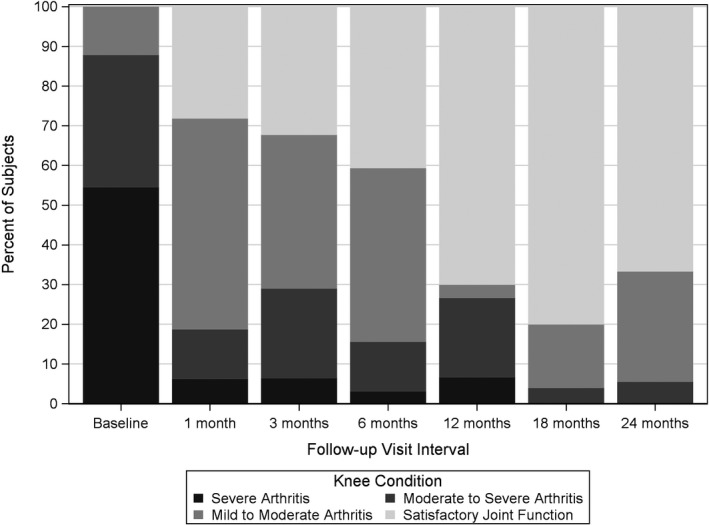
Chronology of Oxford Knee Score study results.

The mean (±SD) OKS scores continued to increase from baseline to 18 months and remained stable at 24 months, landing at 47.2 ± 8.1 and 46.8 ± 10.3, respectively, compared to the baseline score of 20.2 ± 7.3 (*P* < 0.0001) (Table 3). Eighteen of 33 subjects (54.6%) in this subset reported having symptoms consistent with “severe arthritis” at baseline, and this decreased to 0% at 18 and 24 months. Additionally, the functional improvements noted in the first 12 months continued through the 24‐month follow‐up, with 66.7% of those returning at 24 months still indicating “satisfactory joint function” (Figure 4).

**Table 3:** Oxford Knee Score Results

	Baseline *n* = 33^b^	1 month *n* = 32^b^	3 months *n* = 31^b^	6 months *n* = 32^b^	12 months *n* = 30^b^	18 months *n* = 25	24 months *n* = 18
Oxford Knee Score^a^							
Mean ± SD	20.2 ± 7.3	35.0 ± 7.5	34.9 ± 8.6	36.9 ± 8.2	40.6 ± 11.7	47.2 ± 8.1	46.8 ± 10.3
95% CI	17.7 to 22.9	32.3 to 37.7	31.8 to 38.1	34.0 to 39.9	36.2 to 45.0	43.8 to 50.5	41.7 to 52.0
Distribution of Oxford Knee Score classification^a^							
Score 0 to 19 (severe)	18 (54.6)	2 (6.3)	2 (6.5)	1 (3.1)	2 (6.7)	0 (0.0)	0 (0.0)
Score 20 to 29 (moderate to severe)	11 (33.3)	4 (12.5)	7 (22.6)	4 (12.5)	6 (20.0)	1 (4.0)	1 (5.6)
Score 30 to 39 (mild to moderate)	4 (12.1)	17 (53.1)	12 (38.7)	14 (43.8)	1 (3.3)	4 (16.0)	5 (27.8)
Score 40 to 48 (satisfactory function)	0 (0.0)	9 (28.1)	10 (32.3)	13 (40.6)	21 (70.0)	20 (80.0)	12 (66.7)

a Data are presented as mean and standard deviation (SD) along with 95% confidence interval (CI), or number of subjects (%).

b Data from original study were included from baseline to 12 months for subjects enrolled in this extension study.

### Global Perceived Effect

Eighty percent of subjects (20/25) reporting data at 18 months and 66.7% of subjects (12/18) at 24 months reported a perceived improvement in their chronic pain condition following a single treatment with CRFA (Table 4).

**Table 4:** Global Perceived Effect

	1 month *n* = 32^b^	3 months *n* = 31^b^	6 months *n* = 32^b^	12 months *n* = 30^b^	18 months *n* = 25	24 months *n* = 18
Distribution of global perceived effect score^a^						
Not improved/worse	5 (15.6)	8 (25.8)	3 (9.4)	4 (13.3)	5 (20.0)	6 (33.3)
Improved	27 (84.4)	23 (74.2)	29 (90.6)	26 (86.7)	20 (80.0)	12 (66.7)
95% CI (improved)	67.2 to 94.7	55.4 to 88.1	75.0 to 98.0	69.3 to 96.2	59.3 to 93.2	41.0 to 86.7

a Data are presented as number of subjects (%) or 95% exact binomial confidence interval (CI).

b Data from original study were included from 1 to 12 months for subjects enrolled in this extension study.

### Kaplan‐Meier Survivor Analysis

**Figure 5:**
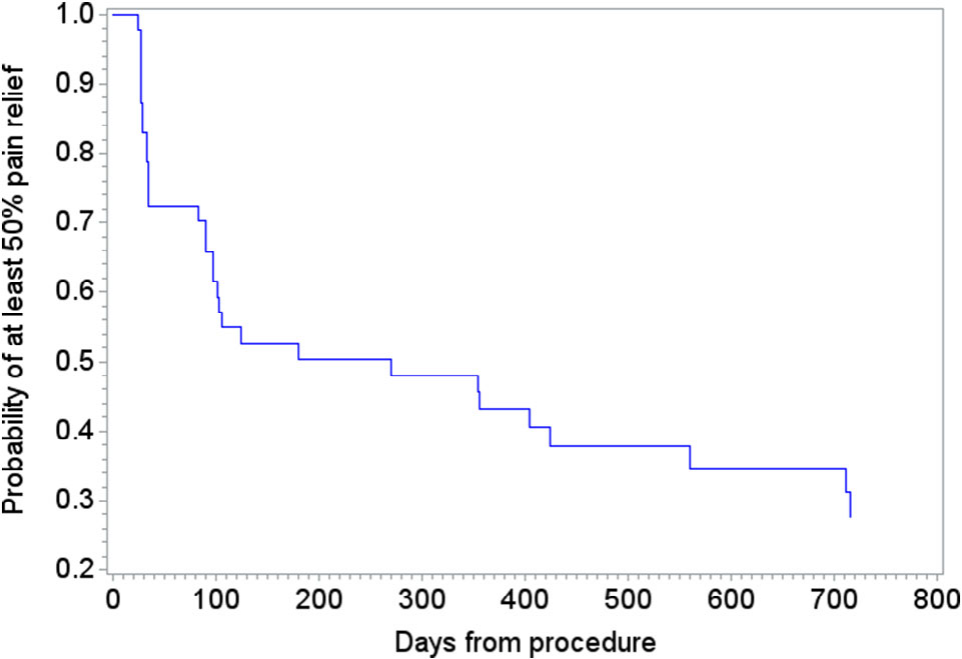
Kaplan‐Meier survivor analysis.

The Kaplan‐Meier survivor curve is depicted in Figure 5. This analysis suggests that the small subset of patients in this study had an approximately 35% chance of maintaining 50% or greater pain relief through 700 days after RFA.

### Radiographic Evaluation

Radiographic data were not required as part of follow‐up in the extension study for subjects completing assessments via telephone; thus, data were extremely limited. However, results from the available images at 24 months demonstrated an improvement of 1 OA grade from baseline in 33.3% of subjects (2/6), no change in 50.0% of subjects (3/6), and worsening of 1 OA grade in 16.7% of subjects (1/6). The results were similar at 18 months, with 33.3% of subjects (4/12) demonstrating an improvement of 1 OA grade, no change in 58.3% of subjects (7/12), and worsening of 1 OA grade in 8.3% of subjects (1/12), suggesting that CRFA does not cause and/or is not associated with joint degeneration over the time periods included in this analysis.

### Adverse Events

There were no serious or nonserious AEs related to the CRFA procedure reported at 18 and 24 months following CRFA. Adverse events reported up to 12 months post‐CRFA were detailed in previous publications.^13^, ^14^

## Discussion

The ability of RFA ablation to reduce pain and improve function in patients with OA of the knee has been well established^1^, ^13^, ^19^, ^20^; however, data on outcomes beyond 12 months have not previously been reported. The majority of publications examining standard RFA (SRFA) for patients with this indication conclude follow‐up at or before 6 months, consistent with the anticipated known effect for SRFA.^19^, ^20^, ^21^ In addition, twelve‐month analgesia utilizing CRFA has been previously described for painful conditions in the back.^6^, ^7^, ^9^, ^10^ Ho et al. previously suggested that CRFA can provide analgesic effect for 24 months when used to treat painful sacroiliac joints, and data from this series continues to support that concept.^8^

Even for those 15 patients in Table 1 who were excluded from this extension study due to the need for a subsequent intervention on their index knee post‐CRFA, the durability of CRFA is identified in that the average number of days from the time of CRFA to the other procedure or injury was 363 days (range 26 to 664 days), with 3 subjects experiencing satisfactory results beyond 18 months post‐CRFA.

Interestingly, the functional improvements identified in the OKS at the 12‐month time point^14^ continued to be present at 24 months in this subset of patients, suggesting that removing a painful stimulus allows increased movement, which in turn improves function of the joint.

The underlying mechanism of analgesic effect behind SRFA and CRFA technologies is thought to be the same; that is, both technologies create a thermal lesion in sensory nerves by channeling focused energy and causing sustained temperatures in excess of 80°C. Peripheral sensory nerve regeneration rates following injury (such as thermal ablation) are well documented.^22^ As described by Rojhani et al.,^23^ the use of COOLIEF* cooled radiofrequency allows greater energy to be applied due to the water‐cooling mechanism, which minimizes temperature extremes at the probe tip and facilitates the creation of a larger lesion. The larger lesion may afford more efficient and longer periods of analgesia by enabling more accurate targeting of culprit nerves and requiring longer duration of nerve healing, respectively.

### Limitations

A limitation of this study was its small sample size, with only a subset of patients enrolled in the trial being included in this analysis (see Figure 2). There are several reasons driving this outcome, including the loss of 2 investigators, participation at only 5 of the original 11 trial sites, the inability to contact 35 of the patients, and patient exclusion due to use of alternate methods for treating their OA knee pain post‐CRFA. Consequently, data from the 2 different CRFA‐treated study populations were combined to facilitate an “*N*” for each outcome at the 18‐month time point from which statistical analyses could be performed. Moreover, because this study was initiated 6 months after the conclusion of the original study, the timing of this latest data analysis contributed to patient attrition. In an attempt to compensate for this timing, wide follow‐up windows of ±3 months were allowed; however, 3 patients reported data beyond the windows for the study. There may also have been a bias towards unwillingness to return for follow‐up, as CRFA does not involve a permanent implant and all patients had been referred to pain physicians for their symptom management. Additionally, the unblinded nature of the trial presents potential for bias.

Despite these limitations, as this is the first publication to present prospectively collected outcomes through 18 and 24 months following any radiofrequency procedure, findings from this study add important data to the pool of literature. The results suggest that COOLIEF* cooled radiofrequency has the capability to provide sustained analgesia and functional improvement up to 24 months after a single application in patients suffering from OA knee pain.

## Conclusions

In this subset of subjects from a randomized controlled study, COOLIEF* CRFA of 3 genicular nerves safely provided sustained pain relief, improved function, and favorable perceived effect through 24 months for patients with chronic OA knee pain.

## Conflicts of Interest

Drs. Davis, DePalma, and Kapural are paid consultants (clinical advisory board) for Avanos Medical, Inc.

## Role of Sponsor

The sponsor, Avanos Medical, Inc., designed the study protocol, operationalized the study (including establishing appropriate controls), and managed the creation of this manuscript by the study investigators. Statistical services were performed by a third party, independently of Avanos Medical, Inc.

## Disclosure

This study was conducted as an extension to a previously conducted study, and the 6‐month results from the previous study were presented as a poster and platform abstract at the American Society of Regional Anesthesia and Pain Medicine 2016 (November) Meeting in San Diego, CA, and the American Academy of Pain Medicine 2017 (March) Meeting in Orlando, FL, and published in manuscript form in *Regional Anesthesia and Pain Medicine* (January 2018). The 12‐month data were presented at the European Society of Regional Anesthesia Congress in 2017 in Lugano, Switzerland, and were published in manuscript form in *Regional Anesthesia and Pain Medicine* (2019).
